# Varicose veins and its risk factors among nurses at Dhulikhel hospital: a cross sectional study

**DOI:** 10.1186/s12912-020-0401-8

**Published:** 2020-02-03

**Authors:** Regan Shakya, Robin Man Karmacharya, Rojina Shrestha, Archana Shrestha

**Affiliations:** 1 Department of Physiotherapy, Kathmandu University School of Medical Sciences/Dhulikhel Hospital, Kavre, Dhulikhel, Nepal; 2 Department of Cardiovascular Surgery, Kathmandu University School of Medical Sciences/Dhulikhel Hospital, Kavre, Dhulikhel, Nepal; 3 Department of Community Programs, Kathmandu University School of Medical Sciences/Dhulikhel Hospital, Kavre, Dhulikhel, Nepal

**Keywords:** Varicose veins, Nurses, Standing position, Risk factors

## Abstract

**Background:**

Women in nursing professions are at high risk for developing varicose veins as it requires physical work and prolonged standing. The aim of the study is to estimate the current prevalence of varicose veins among nurses at Dhulikhel Hospital and assess its risk factors.

**Methods:**

A cross sectional study was carried out among 181 female nurses from different clinical settings of Dhulikhel Hospital. A structured questionnaire was administered to gather the demographic, work related and medical history information. The participants underwent Doppler ultrasound for varicose veins confirmation. Varicose veins was defined as Doppler finding of reflux or vein diameter equal or greater than 5 mm.

**Results:**

A total of 181 nurses participated in this study and 83 (46%) had varicose veins. The mean standing time was 4.28 (0.8) hours /day, mean sitting time was 1.28 (0.6) hours/day, mean walking time was 2.37 (0.8) hours/day. In the adjusted model the odds of having varicose veins was 27 times greater with every 1 hour increase in standing time per day (adjusted OR: 27.44; 95% CI 4.09–180.77; *p*-value <0.00).

**Conclusions:**

Varicose veins was prevalent among nurses’ at Dhulikhel Hospital. Prolonged standing was found to be a significant factor for varicose veins.

## Background

Varicose veins are a common chronic venous disorder affecting 20 to 60% of adults worldwide [1–4]. However, the disease occurrence varies significantly by geographical region; with comparatively lowered incidence of 19% in Asian ethnic group than the other ethnic groups [5, 6]. Varicose veins are often believed to be a cosmetic problem. However, they may cause serious complication including pain, discomfort, leg cramps, ulceration, poor quality of life, absenteeism, and even loss of life [7–10]. Women have 2–3 times higher risk of having varicose veins than men [4, 11]. Longitudinal studies have suggested that occupations requiring prolonged standing increase the risk of surgery and subsequent hospitalization for varicose veins [12–14]. Multiple cross sectional studies have identified strong positive correlations between prolonged standing at work and varicose veins [9, 15].

Women in nursing professions are at high risk for developing varicose veins as their job requires physical work and prolonged standing [16]. Although multiple factors have been identified as risks for varicose veins, there is limited information on its prevalence and risk factor among nurses in Nepal. Therefore, we aim to estimate the current prevalence of varicose veins among nurses of Dhulikhel Hospital and assess the risk factors.

## Methods

### Study design and setting

This cross sectional study was conducted between November 2017 and February 2018 at Dhulikhel Hospital, a Kathmandu University Hospital.

### Study participants

We recruited 181 nurses with minimum of 6 months of clinical experience. The participants were all female and included subjects from different clinical work stations of the hospital (gynecology ward, pediatrics ward, operating theatres, surgery ward, orthopedics ward, critical care units, medicine ward, out-patient departments (OPD), and teaching faculties). We excluded anyone if they (a) had surgeries or anesthesia within the past 6 months, (b) neurological conditions, (c) were currently pregnant or pregnant in the past year, and (d) a history of varicose veins prior to starting nursing practice. Prior to the study, we provided the information regarding the study to the participants and obtained their written consent.

### Data collection

We administered a structured questionnaire to measure:

1. The demographic information such as age (in years), education (certificate level/ bachelors or above) marital status (married/ not married), weight (in kilograms), height (in meters), Body Mass Index (BMI), parity (nulliparous/ parous).
2. Work related information including clinical experience (in years), clinical work stations, time spent in standing (hours/ day), time spent in sitting (hours/ day) and time spent in walking (hours/ day).
3. Medical history including family history of varicose veins (present/ absent), Bowel movements (Regular/ irregular).

### Outcome

The primary outcome was varicose veins. We defined varicose veins based on the Comprehensive classification system for chronic venous disorders (CEAP) [17]. A specialist cardiovascular surgeon clinically examined the participants in the seven clinical classes of CEAP ranging from C0 to C6. Those categorized as higher than C0 are clinically suspected of having varicose veins. All the participants underwent a Doppler examination using Siemens- Acuson P300 doppler ultrasound with linear probe 7–12 MHz. The participants stood on a ‘Doppler step’ - a square platform of 24 in. length and 20 in. height from the ground during the Doppler procedure. The Doppler findings of reflux or vein diameter equal or greater than 5 mm was confirmed as varicose veins [18, 19].

### Statistical analysis

We summarized the sample characteristics using mean (standard deviation (SD)) for continuous variables and frequency (percentage) for categorical variable. We assessed the association between varicose veins and the nurses’ clinical work stations using chi-square test. We utilized multivariate logistic regression model to assess the association of varicose veins with age, BMI, marital status, parity, education level, bowel habit, family history, work experience, time spent in standing, time spent in sitting and time spent in walking. We presented both crude and adjusted odds ratio (OR) with 95% confidence interval (CI) and *p*-value. We also conducted the analysis for walking hours with categorical variables (<= 3 h per day/ > 3 h per day) to make it comparable to other studies [Data not shown]. We used SPSS version 21 for the analysis.

## Results

Table 1 describes the characteristics of the 181 participant nurses by their status of varicose veins. The mean age of the participants was 26 years. All of them were female. Approximately a third of them were married and 16% had at least one child. About one-fifth reported to have a family history of varicose veins. More than half of the nurses spent their working hours standing. The mean standing time per day was 4 hours and the mean sitting time was 2 hours. The mean BMI was 24 kg/ m^2^.

**Table 1:** Characteristics of study participants

Characteristics	Frequency (%)		
	Participants with varicose veins^a^ (*n* = 83)	Participants without varicose veins (*n* = 98)	Total Participants (*n* = 181)
Age (years), mean (SD), [range]	26.8 (6.8) [19–52]	24.6 (5.5) [19–56]	25.6 (6.2) [19–56]
BMI (kg/ m^2^), mean (SD), [range]	24.36 (4.7) [15.9–40.8]	23.01 (4.6) [16–43.3]	23.63 (4.7) [15.9–43.3]
Work station			
Gynecology ward	7 (8.4)	10 (10.2)	17 (9.4)
Pediatrics ward	9 (10.8)	16 (16.3)	25 (13.8)
Operating theatres	14 (16.9)	18 (18.4)	32 (17.7)
Surgery ward	7 (8.4)	6 (6.1)	13 (7.2)
Orthopedics ward	5 (6.0)	12 (12.2)	17 (9.4)
Critical care units	14 (16.9)	10 (10.2)	24 (13.3)
Medicine ward	10 (12.0)	17 (17.3)	27 (14.9)
Out-patient department	7 (8.4)	6 (6.1)	13 (7.2)
Teaching faculty	10 (12.0)	3 (3.1)	13 (7.2)
Marital status			
Married	31 (53.4)	27 (46.6)	58 (32.0)
Unmarried	52 (42.3)	71 (57.7)	123 (68.0)
Parity			
Nulliparous	64 (42.2)	87 (57.6)	151 (83.4)
Parous	19 (63.3)	11 (36.7)	30 (16.6)
Education			
Certificate level	59 (41.5)	83 (58.5)	142 (78.5)
Bachelors or above	24 (61.5)	15 (38.5)	39 (21.5)
Bowel habit			
Regular	69 (43.7)	89 (56.3)	158 (87.3)
Irregular	14 (60.9)	9 (39.1)	23 (12.7)
Family history			
Present	23 (69.7)	10 (30.3)	33 (18.2)
Absent	60 (40.5)	88 (59.5)	148 (81.8)
Work experience (in years), mean (SD), [range]	5.23 (6.1) [1–28]	3.89 (4.9) [1–35]	4.5 (5.4) [1–35]
Time spent in standing (hours/day), mean (SD), [range]	3.28 (0.8) 3.29 [3 - 6]	3.02 (0.9) [1–6]	3.60 (1.0) [1–6]
Time spent in sitting (hours/ day), mean (SD), [range]	1.28 (0.6) [1–4]	1.86 (0.8) [1–4]	1.59 (0.8) [1–4]
Time spent in walking (hours/ day), mean (SD), [range]	2.37 (0.8) [1–4]	3.03 (0.9) [1–5]	2.72 (0.9) [1–5]

^a^The Doppler findings of reflux or vein diameter equal or greater than 5 mm

**Fig. 1:**
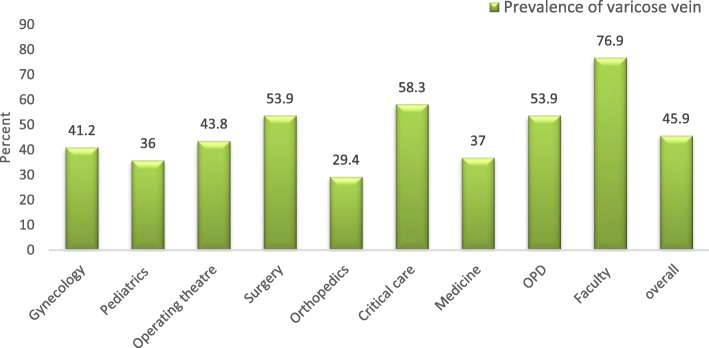
Prevalence of varicose veins* of nurses among all clinical work stations (*p* < 0.01). *The Doppler findings of reflux or vein diameter equal or greater than 5 mm

**Fig. 2:**
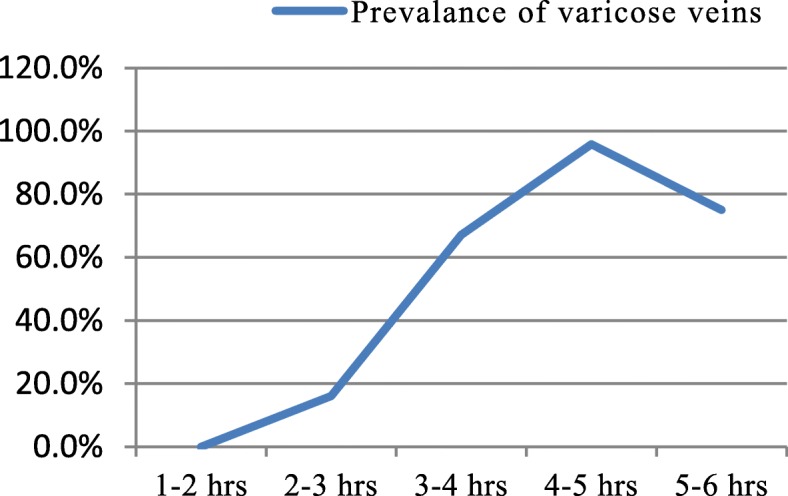
Prevalence of varicose veins among nurses with hours spend standing per day. (p-trend < 0.00)

Nearly three-quarters of the participants had no visible veins present. One-fifth of the participants had a clinical presence of edema at their lower extremity (Table 2). The overall prevalence of varicose veins among the nurses at Dhulikhel Hospital was 46% (Fig. 1). There was statistically significant difference in prevalence of varicose veins by the clinical work stations (*p* = 0.01). The highest prevalence was among teaching faculties who were actively engaged in teaching and the lowest was in subjects in the orthopedic ward. Out-patient departments, surgery ward, and critical care units also had higher prevalence compared to pediatrics ward, medicine ward and orthopedics ward. Figure 2 displays the prevalence of varicose veins among the nurses within each hour spent standing per day (dose response relationship). The prevalence is markedly increased in the nurses with long standing hours. (Fig. 2).

**Table 2:** Distribution of clinical classification of varicose veins (*n* = 181)

Clinical classification of CEAP	Frequency (%)
C0: No visible veins	131 (72.4%)
C1: Reticular veins	5 (2.8%)
C2: Varicose veins	7 (3.9%)
C3: Edema	33 (18.2%)
C4a: Pigmentation or eczema	5 (2.8%)
C4b: Lipodermatosclerosis	0 (0%)
C5: Healed venous ulcer	0 (0%)
C6: Active venous ulcer	0 (0%)

Table 3 presents the factors associated with varicose veins among the participant nurses. The odds of having varicose veins is 27 times greater with every 1 hour increase in standing time after adjusting for age, BMI, marital status, parity, education, bowel habit, family history, work experience, time spent on standing, time spent on sitting and time spent on walking (adjusted OR: 27.44; 95% CI 4.09–180.77; *p*-value <0.00). In the unadjusted model, age, parity, education and family history were positively associated with varicose veins but the association was not significant after adjusting for socio-demographic variables and working hours’ pattern. Sitting hours and walking hours were negatively associated with varicose veins prevalence. However, these associations were not significant in multivariate model (Table 3). In the multivariate model, when categorizing the time spent standing, the odds of varicose veins were nine times higher among those who reported to stand 3 hours or more compared to those who stood less than 3 hours after adjusting to the variables. (adjusted OR: 8.8; 95% CI 2.2–35.8; *p*-value <0.001) (Additional file 1: Table S1).

**Table 3:** Factors associated with varicose veins among nurses at Dhulikhel Hospital (*n* = 181)

Factors	Unadjusted Odds Ratio *n* = 181			Adjusted Odds Ratio *n* = 181		
	OR	95% CI	*P* value	OR	95% CI	*P* value
Age (years)	**1.06**	**1.01–1.12**	**0.03**	1.06	0.90–1.25	0.47
BMI (kg/ m^2^)	1.07	1.00–1.14	0.06	1.10	0.99–1.22	0.07
Marital status						
Unmarried	Ref			Ref		
Married	1.57	0.84–2.94	0.16	0.44	0.13–1.55	0.20
Parity						
Nulliparous	**Ref**			Ref		
Parous	**2.35**	**1.05–5.28**	**0.04**	3.78	0.65–22.17	0.14
Education						
Certificate level	**Ref**			Ref		
Bachelors or above	**2.25**	**1.09–4.65**	**0.03**	3.12	0.90–10.77	0.07
Bowel habit						
Regular	Ref			Ref		
Irregular	2.01	0.82–4.91	0.13	1.74	0.49–6.10	0.39
Family history						
Absent	**Ref**			Ref		
Present	**3.37**	**1.50–7.60**	**<0.00**	1.41	0.48–4.12	0.53
Work experience (in years)	1.05	0.99–1.11	0.11	0.88	0.73–1.05	0.16
Time spent in standing (hours/day)	**6.84**	**3.85–12.18**	**<0.00**	**27.44**	**4.09–180.77**	**<0.00**
Time spent in sitting (hours/ day)	**0.31**	**0.19–0.52**	**<0.00**	2.60	0.48–14.04	0.27
Time spent in walking (hours/day)	**0.40**	**0.27–0.60**	**<0.00**	4.16	0.71–24.24	0.11

Statistically significant results are presented in bold (*p* < 0.05)

## Discussion

Nearly half of the practicing nurses at Dhulikhel Hospital have varicose veins. In our study, those having to stand for longer period of time at work had higher chances of having varicose veins. The prevalence of varicose veins was highest among nurses who stood for 4–5 h a day. Among the nurses, the teaching faculty had the highest varicose veins prevalence. It could be attributed to common practice of standing while teaching and their clinical duties. Similarly nurses from critical care, surgery and out-patient departments were also among the most affected which could be due to relative differences in standing hour compared to sitting hours. Tuchsen et al. reported the relative risk of 1.8 for developing varicose veins in occupations requiring higher proportion of standing work in compared to sitting work [13].

In this study, 46% of the nurses were affected with varicose veins. As expected, the prevalence is consistent with the studies from general populations that have reported the prevalence of varicose veins from 20 to 60% [1–4]. Compared to our study, higher prevalence was reported among the nurses’ population from Iran [16, 20]. However, a study from India reported a lower prevalence amongst the nurses at 24% [21]. The variation in the prevalence among nurses might be attributable to differences in the working environment and clinical settings. For example, 57% had increased standing hours in one duty shift in India [21], three quarters of the nurses in Iran had standing period for more than 2 hours and nearly 40% of them have more than 4 hours standing time per day [16]. Similarly 48% prevalence of varicose veins was observed among hairdressers in Iran with an average of more than 3 hours standing at work [20].

In our study we found that standing hours was positively associated with varicose veins; resulting in almost 27 times increased odds with every 1 hour increase in standing. There was linear- dose response relationship between the standing hours and prevalence of varicose veins, which explains the effect of prolonged standing in acquiring varicose veins. The probability of developing varicose veins was almost certain in nurses who stand daily for 4–5 h. The result showed prolonged standing as a major risk factor for varicose vein. Krijnen et al. reported standing position as an aggravating factor for varicose veins in the European population [22]. A study among the hair dressers in Taiwan reported that the odds ratio of having varicose veins was 32 for those standing more than 260 h a month [23]. After categorizing the standing hours, our study reported a nine times increased odds of varicose veins for standing for more than 3 hours per day compared to those standing less than 3 hours a day. This odds ratio is higher compared to the global scale. Globally, the studies have reported odds of varicose veins of 2 to 3 times for standing more than 3 hours a day compared to standing less than 3 hours a day [15, 20, 24]. A study among nurses in Iran reported four times increased odds of having varicose veins for those standing more than 4 hours compared to those who stand for a lesser time [16]. Prolonged standing facilitates the development of varicose veins. Physiologically standing has a strong negative effect on venous return. With prolonged standing the increase in venous pressure markedly affects the one way valves of the lower extremities. This ultimately weakens or damages the valve which compromises the hemodynamics and increases venous stasis leading to varicose veins via vein enlargement or blood reflux [25].

In our study, varicose veins was associated with age, parity, level of education and family history in an unadjusted model. Other studies have reported obesity, pregnancy and family history as the major risk factors for varicose veins [3, 4, 11, 26, 27]. After adjusting for other variables, our study didn’t show the association of age, parity, level of education and family history with varicose veins. Our result for pregnancy and obesity is similar with other studies with multivariate analysis [20, 23]. Some of the risk factors might have been the aggravating factor rather than primary causative factor [20]. Similarly, increased sitting time and walking time showed a beneficial effect on varicose veins in the unadjusted model; which could suggest that sitting and walking can assist to relieve the varicose veins but not prevent from the disease formation.

### Strength and limitations

Our study has several strengths. First, the quality of data regarding varicose veins was high. All participants underwent Doppler ultrasound, an objective measure to determine their vascular condition. The assessment was performed by an expert cardiovascular surgeon with more than 5 years of experience in the related field. Some participants with clinically “no visible veins” (CEAP: C0) have been diagnosed with varicose veins when assessed with Doppler ultrasound. Secondly, we were able to control multiple confounding factors in our analysis by using multivariate logistic regression.

Our study is not devoid of limitations. Since this is a cross sectional study, both risk factors and varicose veins were measured at one point of time therefore, causality cannot be established. The measurement of hours spent in standing, sitting or walking was self-reported thus the participants might have underestimated or overestimated their standing hours, especially if they had known about their disease condition. This study was conducted among nurses of one center (hospital), so the results might not be generalized to other population.

## Conclusions

Varicose veins were predominantly present with a prevalence of 46% among the nurses at Dhulikhel Hospital. Prolonged standing was found to be a significant risk factor for varicose veins. The nurses should try to avoid unnecessary prolonged standing at work and use sitting or walking whenever possible. This change in clinical practice could ultimately reduce the risk for vascular disease.

## Supplementary information

**Additional file 1: Table S1.** Factors associated with varicose vein among nurses at Dhulikhel Hospital (*n* = 181).

## Data Availability

The dataset generated and/or analyzed during the current study are available from the corresponding author on a reasonable request.

## Abbreviations

BMI: Body Mass Index
CEAP: Comprehensive classification system for chronic venous disorders (C: Clinical, E: Etiologic, A: Anatomic, P: Pathophysiologic)
CI: Confidence Interval
MHz: Megahertz
OPD: Outpatient department
OR: Odds Ratio
SD: Standard Deviation
SPSS: Statistical Package for the Social Sciences
