# Supplementary material for: Varicose veins and its risk factors among nurses at Dhulikhel hospital: a cross sectional study
Source: BMC Nurs. 2020 Feb 3;19:8. doi: 10.1186/s12912-020-0401-8 (PMC6998362; doi:10.1186/s12912-020-0401-8)
Supplement: Supplementary file 1 — Additional file 1: Table S1. Factors associated with varicose vein among nurses at Dhulikhel Hospital (n = 181). [file 12912_2020_401_MOESM1_ESM.docx]

Table S1 Factors associated with varicose vein among nurses at Dhulikhel Hospital (n=181)

|  | | | | | | |
| --- | --- | --- | --- | --- | --- | --- |
| Factors | Unadjusted Odds Ratio  n =181 | | | Adjusted Odds Ratio  n=181 | | |
|  | OR | 95% CI | P value | OR | 95% CI | P value |
| Age (years) | **1.06** | **1.01 – 1.12** | **0.03** | 1.06 | 0.90 – 1.24 | 0.48 |
| BMI (kg/ m2) | 1.07 | 1.00 – 1.14 | 0.06 | 1.08 | 0.98 – 1.20 | 0.11 |
| Marital status  Unmarried  Married | Ref  1.57 | 0.84 – 2.94 | 0.16 | 0.72 | 0.22 – 2.37 | 0.58 |
| Parity  Nulliparous  Parous | **Ref**  **2.35** | **1.05 – 5.28** | **0.04** | 2.62 | 0.48 – 14.34 | 0.27 |
| Education  PCL  Bachelors or above | **Ref**  **2.25** | **1.09 – 4.65** | **0.03** | 2.90 | 0.84 – 10.04 | 0.09 |
| Bowel habit  Regular  Irregular | Ref  2.01 | 0.82 – 4.91 | 0.13 | 1.49 | 0.44 – 5.09 | 0.53 |
| Family history of varicose vein  Absent  present | **Ref**  **3.37** | **1.50 – 7.60** | **0.00** | 1.47 | 0.51 – 4.20 | 0.47 |
| Work experience (in years) | 1.05 | 0.99 – 1.11 | 0.11 | 0.90 | 0.75 – 1.07 | 0.22 |
| **Standing hour (hours/ day)**  **<= 3 hours/day**  **> 3 hours/day** | **Ref**  **24.01** | **10.49 – 54.93** | **0.00** | **8.81** | **2.18 – 35.65** | **<0.00** |
| **Sitting hours (hours/ day)** | **0.314** | **0.19 – 0.52** | **0.00** | **0.40** | **0.18 – 0.90** | **0.03** |
| Walking hours (hours/day) | **0.40** | **0.27 – 0.60** | **0.00** | 0.58 | 0.26 – 1.28 | 0.18 |
| Statistically significant results are presented in bold (p < 0.05) | | | | | | |
